# Supplementary material for: Alterations in the amplitude and burst rate of beta oscillations impair reward-dependent motor learning in anxiety
Source: eLife. 2020 May 19;9:e50654. doi: 10.7554/eLife.50654 (PMC7237220; doi:10.7554/eLife.50654)
Supplement: Transparent reporting form [file elife-50654-transrepform.pdf]

eLIFE
1st Floor
24 Hills Road
Cambridge CB2 1JP, UK
P 01223 855340
W elifesciences.org
T @elife

eLife's transparent reporting form

We encourage authors to provide detailed information *within their submission* to facilitate the interpretation and replication of experiments. Authors can upload supporting documentation to indicate the use of appropriate reporting guidelines for health-related research (see EQUATOR Network), life science research (see the BioSharing Information Resource), or the ARRIVE guidelines for reporting work involving animal research. Where applicable, authors should refer to any relevant reporting standards documents in this form.

If you have any questions, please consult our Journal Policies and/or contact us: editorial@elifesciences.org.

Sample-size estimation

- You should state whether an appropriate sample size was computed when the study was being designed
- You should state the statistical method of sample size computation and any required assumptions
- If no explicit power analysis was used, you should describe how you decided what sample (replicate) size (number) to use

Please outline where this information can be found within the submission (e.g., sections or figure legends), or explain why this information doesn't apply to your submission:

See section **Methods and Materials**, subsection "**Participants and sample size estimation**"

Replicates

- You should report how often each experiment was performed
- You should include a definition of biological versus technical replication
- The data obtained should be provided and sufficient information should be provided to indicate the number of independent biological and/or technical replicates
- If you encountered any outliers, you should describe how these were handled
- Criteria for exclusion/inclusion of data should be clearly stated
- High-throughput sequence data should be uploaded before submission, with a private link for reviewers provided (these are available from both GEO and ArrayExpress)

Please outline where this information can be found within the submission (e.g., sections or figure legends), or explain why this information doesn't apply to your submission:

eLife Sciences Publications, Ltd is a limited liability non-profit non-stock corporation incorporated in the State of Delaware, USA, with company number 5030732, and is registered in the UK with company number FC030576 and branch number BR015634 at the address 1st Floor, 24 Hills Road, Cambridge CB2 1JP | August 2014

We describe one main EEG + behavioral experiment and one additional behavioral experiment. See section **Methods and Materials**, and subsections "**Participants and sample size estimation**"; section Behavioral Results, subsection "State anxiety during reward-based learning reduces learning rates if there is no prior baseline exploration", Legend of **Figure 4, Figure 6.** While the second control experiment confirms and replicates some of the findings from the main experiment, it was designed to provide new evidence to help us interpret the data from the main experiment (e.g. what would be the effect of the anxiety manipulation if there is not a baseline exploration phase?) In the manuscript here is not a replication of the same experiment.

Statistical reporting

- Statistical analysis methods should be described and justified
- Raw data should be presented in figures whenever informative to do so (typically when N per group is less than 10)
- For each experiment, you should identify the statistical tests used, exact values of N, definitions of center, methods of multiple test correction, and dispersion and precision measures (e.g., mean, median, SD, SEM, confidence intervals; and, for the major substantive results, a measure of effect size (e.g., Pearson's r, Cohen's d)
- Report exact p-values wherever possible alongside the summary statistics and 95% confidence intervals. These should be reported for all key questions and not only when the p-value is less than 0.05.

Please outline where this information can be found within the submission (e.g., sections or figure legends), or explain why this information doesn't apply to your submission:

See section **Methods and Materials**, and subsection "Statistical Analysis". We provide details on the statistical tests applied, the exact values of N, the dispersion measures (SEM) for the mean population values, the method used to control for multiple comparisons, and also a non-parametric effect size estimator and its confidence intervals. When applying control for multiple comparisons using the false discovery rate, we write " $P_{FDR} < 0.05$" as used in previous eLife papers.

(For large datasets, or papers with a very large number of statistical tests, you may upload a single table file with tests, Ns, etc., with reference to sections in the manuscript.)

Group allocation

- Indicate how samples were allocated into experimental groups (in the case of clinical studies, please specify allocation to treatment method); if randomization was used, please also state if restricted randomization was applied
- Indicate if masking was used during group allocation, data collection and/or data analysis

Please outline where this information can be found within the submission (e.g., sections or figure legends), or explain why this information doesn't apply to your submission:

See section **Results.** Participants were pseudo-randomly allocated to either an experimental or control group.

Additional data files ("source data")

- We encourage you to upload relevant additional data files, such as numerical data that are represented as a graph in a figure, or as a summary table

- Where provided, these should be in the most useful format, and they can be uploaded as "Source data" files linked to a main figure or table
- Include model definition files including the full list of parameters used
- Include code used for data analysis (e.g., R, MatLab)
- Avoid stating that data files are "available upon request"

Please indicate the figures or tables for which source data files have been provided:

Raw Data files are available in the Open Science Framework Data Repository:
https://osf.io/mfe2j/
These data corresponds to analyses in Figures 2-11.
